# *Bacillus pumilus* SG44 Reshapes the Microbiome and Alters Metamorphosis and Development of *Aurelia coerulea* Polyps

**DOI:** 10.3390/microorganisms14102338

**Published:** 2026-10-13

**Authors:** Min Li, Xiangrui Guo, Manzoor Ahmad, Jiajing Yang, Jie Sun, Zirui Li, Hongyuan Lin, Yuting Zhang, Ye Zhao, Hao Sun, Yanying Zhang

**Affiliations:** 1 School of Ocean, Yantai University, Yantai 264005, China; lmin1821@s.ytu.edu.cn (M.L.);; 2 Department of Integrative Agriculture, College of Agriculture and Veterinary Medicine, United Arab Emirates University, Al Ain 200120, United Arab Emirates; 3 Fisheries College, Ocean University of China, Qingdao 266003, China; 4 College of Marine Sciences, South China Agricultural University, Guangzhou 510642, China

**Keywords:** *Aurelia coerulea*, *Bacillus*, settlement, metamorphosis, microbiome

## Abstract

Planula settlement and metamorphosis are critical transitions in the life cycle of the moon jellyfish *Aurelia coerulea*, and associated microorganisms can influence these processes. We developed a co-culture system combining the marine bacterium *Bacillus pumilus* SG44 with *A. coerulea* planula larvae and, using 16S rRNA gene high-throughput sequencing, examined how strain SG44 affects larval settlement and metamorphosis and the associated microbiota. Treatment with strain SG44 significantly elevated the settlement and metamorphosis rate of planulae, although the resulting polyps exhibited morphological deformities. Strain SG44 became established as the dominant population in both polyp tissues and the surrounding culture seawater. Strain SG44 significantly decreased the diversity of the polyp-associated microbial community and markedly reduced the relative abundance of *Neptuniibacter*, a reported beneficial taxon in jellyfish-associated microbiota, as well as other putatively beneficial genera, including *Halomonas* and *Sulfitobacter*. Co-occurrence network analysis revealed that the network of treatment with strain SG44 contracted in size while maintaining a comparably high average degree. These results indicate that strain SG44 affects the development of *A. coerulea* larvae, an effect that may be linked to shifts in the host-associated microbial community, although a causal relationship remains to be established, and provide a new perspective on microbe–host interactions during scyphozoan early development.

## 1. Introduction

*Aurelia coerulea* is widely distributed in the coastal waters of warm-temperate and temperate zones worldwide and is a dominant species in the coastal waters of the Yellow Sea and Bohai Sea in China [1,2]. Its unique reproductive strategies, complex life cycle, and broad tolerance to varying environmental conditions enable it to thrive in marine ecosystems [3,4], and its broad feeding spectrum further contributes to its ecological significance [5]. These resilient biological characteristics, in conducive environmental settings, frequently drive substantial population increases, initiating worldwide jellyfish blooms that present a pervasive ecological challenge in nearshore environments [6]. Jellyfish blooms not only alter food web structure through top-down and bottom-up effects, but also significantly impact marine economic activities such as fisheries, tourism, and the cooling systems of nuclear power plants [7,8]. Currently, mitigation strategies rely primarily on physical and chemical methods, both of which have significant limitations: physical removal is effective only in the short term and at local scales, whereas chemical approaches pose potential ecological risks to non-target organisms [9,10,11,12]. Thus, given the increasingly severe ecological and economic impacts of jellyfish blooms and the inadequacies of conventional control measures, a novel, environmentally friendly intervention is urgently needed.

Marine microorganisms possess diverse metabolic pathways, which play a vital role in marine biogeochemical cycles [13]. Previous studies have shown that distinct microbial assemblages are associated with different locations and life stages of marine invertebrates [14]. Microbes establish parasitic, symbiotic, mutualistic, and even pathogenic relationships with their hosts [15], often participating in host material cycling, vitamin synthesis, and natural product synthesis through “microbe–host” interaction networks [16,17]. The composition of the host and its microbiota, and the connections between them, have a profound impact on biological processes such as the development and maturation of the host [18]. In contrast, microbial communities influence hosts in multiple ways, such as metabolism, developmental regulation, and adaptation to the environment, thereby contributing to host health and fitness [16,19]. For example, *Wolbachia* is required for establishing anterior–posterior polarity and the first cleavage in *Brugia malayi* embryos and can induce widespread apoptosis in filarial nematode larvae, leading to developmental arrest and inhibited molting [20,21].

Based on the work of Weiland-Bräuer, microbial community composition differs between the various life history stages of the jellyfish, and the microbiota also differs between the various somatic regions of the adult jellyfish [3]. Previous studies suggest that the native microbiota of *Aurelia* may contribute to normal asexual reproduction and development [22]. It has been shown that the associated microbiome influences the retinoic acid (RA) signaling pathway of the host, which controls the process of strobilation in *A. coerulea* [4]. Moreover, the absence of the native microbiota of the jellyfish results in significantly impaired development and differentiation of the cnidocytes of the host [23,24]. These findings collectively demonstrate that microorganisms play an important role in supporting key developmental and reproductive functions in *A. coerulea*. Current research on *A. coerulea* and associated microbes has been limited to adult medusae and the transition from polyp to ephyra [22,23,24]. Metamorphosis, which marks the transition from sexual reproduction in the planula stage to asexual reproduction in the polyp stage, is a crucial milestone in the life history of *A. coerulea* and one of the most important recruitment steps for scyphozoan individuals [25]. It is still unclear how microbes mediate settlement and metamorphosis in *A. coerulea* larvae.

To address this knowledge gap, our team previously isolated and purified a collection of bacterial strains from various marine organisms and their habitats. Among these strains, strain SG44, originally isolated from the rhizosphere sediment of *Zostera marina* and identified as *Bacillus pumilus* based on 16S rRNA gene sequencing, attracted our attention because co-culture experiments revealed that it significantly altered the settlement and metamorphosis process of *A. coerulea* planula larvae. Moreover, some *Bacillus* strains, renowned for their diverse bioactive metabolites, have been reported to induce or interfere with larval settlement and metamorphosis in marine invertebrates [26]. We therefore hypothesize that strain SG44 may also mediate the settlement and metamorphosis of *A. coerulea* planula larvae through specific mechanisms. Investigating this phenomenon may help us better understand how microbes function during the settlement and metamorphosis of jellyfish planula larvae. In this study, using a co-culture system of strain SG44 and *A. coerulea* planula larvae, combined with high-throughput microbiome profiling, we investigated the impact of this bacterium on larval settlement and metamorphosis, and examined whether these effects are accompanied by alterations in the host-associated microbial community.

## 2. Material and Methods

### 2.1. Collection of A. coerulea Larvae and Preparation of Strain SG44

The *A. coerulea* larvae used in this study were collected from the Yangma Island area, Yantai City, Shandong Province, China, in July 2024. Sexually mature female *A. coerulea* medusae (*n* = 10), characterized by fully developed gonads with visible planulae in the oral arms, were collected from the coastal waters using a hand net and transferred into a 20-L aquarium containing seawater from their native habitat (temperature 18–21 °C, salinity ~29‰). Only medusae with intact bells and active swimming behavior were selected for the experiment. After acclimatization for 2–4 h at 19 °C under natural light conditions without feeding, newly released planulae were collected by filtration through a 40-mesh sieve, rinsed with sterile seawater, and transferred to a beaker.

Strain SG44 was isolated from the rhizosphere sediment of *Zostera marina* and preserved at the Marine Microecology Laboratory, School of Ocean, Yantai University. From a phylogenetic perspective, strain SG44 is classified within the phylum Firmicutes, class Bacilli, order Bacillales, family Bacillaceae, and genus *Bacillus*, specifically identified as *Bacillus pumilus*. The strain was cultivated on 2216E agar plates. A single colony was inoculated into 200 mL of 2216E liquid medium (Qiujing, Shanghai, China) in a 500 mL conical flask and incubated at 28 °C with shaking at 180 rpm for 48 h. Cells were harvested by centrifugation at 8000× *g* for 15 min at 4 °C, washed twice, and resuspended in sterile seawater. The bacterial concentration was determined by direct counting using a hemocytometer under a light microscope (Leica DM1000 LED, Wetzlar, Germany), and the suspension was adjusted to 2 × 10^7^ cells/mL with sterile seawater for subsequent co-culture experiments.

Larvae were randomly assigned to two experimental regimes: the Control Group, which received an equal volume of sterile seawater, and the SG44 Group, in which strain SG44 was added to achieve a final concentration of 2 × 10^7^ cells/mL. Two parallel culture systems were established for different purposes. Planulae were aseptically transferred into 6-well plates using sterile capillary pipettes, with 10 larvae per well, in two groups, namely the Control Group and SG44 Group, for morphological observation and metamorphosis assessment. Each biological replicate consisted of two 6-well plates containing 120 larvae in total (12 plates per group and 24 plates in the entire experiment). Settlement and metamorphosis were assessed jointly as the proportion of larvae that had settled and completed metamorphosis into polyps. To obtain sufficient biomass for subsequent microbiome sequencing, larvae were cultured in parallel in 1000 mL sterile glass beakers, with each beaker containing 500 larvae in 500 mL of the corresponding culture medium; each beaker served as one biological replicate (six per group; Table S1).

All co-culture experiments were conducted in the dark at 19 °C for 144 h. Dark conditions were selected based on previous reports that planula larvae of scyphozoans exhibit negative phototaxis and naturally prefer to settle on the undersides of substrates or in shaded areas [27,28]. Dark conditions therefore mimic the low-light microenvironments in which settlement naturally occurs. Exogenous food was not introduced to avoid bringing in additional microorganisms or organic matter that could confound the effects of strain SG44 on larval settlement and the subsequent microbial community analysis. Physical disturbance was kept to a minimum to avoid stress. Larval morphology and developmental progression were recorded every 48 h using a stereomicroscope (Leica S9D, Wetzlar, Germany). At the end of the experiment, polyps were imaged, and body length, body width, and tentacle length were measured for at least 30 individuals per group using image-analysis software ImageView (version 4.11.18709, ToupTek Photonics, Hangzhou, China).

### 2.2. Sample Collection

Prior to co-culture, newly released planulae and their surrounding seawater were collected as baseline references, designated as planula tissue (PT) and planula seawater (PW), respectively. After 144 h (6 days) of incubation, metamorphosed polyps from the control group (C) and the treatment with strain SG44 (T) were collected. Approximately 50 mg of polyp tissue was aseptically dissected from each of the six biological replicates per group. Polyps from different biological replicates were not pooled, thereby preserving the independence of each replicate. These samples were labeled as control polyp tissue (CT) and treatment polyp tissue (TT) for subsequent microbial DNA extraction. Immediately following tissue collection, 200 mL of culture seawater was collected from each replicate. Water was filtered through sterile 0.22 μm polycarbonate membranes (Merck Millipore, Darmstadt, Germany), and the filters retaining microbial biomass were collected as control seawater (CW) and treatment seawater (TW). All samples (PT, PW, CT, CW, TT, TW; *n* = 6) were immediately flash-frozen in liquid nitrogen and stored at −80 °C until nucleic acid extraction (Table S1).

### 2.3. DNA Extraction and PCR Amplification

Total genomic DNA was extracted from jellyfish larval and culture seawater samples using the E.Z.N.A.^®^ Soil DNA Kit (Omega Bio-Tek, Norcross, GA, USA). The V3-V4 hypervariable regions of the bacterial 16S rRNA gene were amplified using the primers 338F (5′-ACTCCTACGGGAGGCAGCAG-3′) and 806R (5′-GGACTACHVGGGTWTCTAAT-3′).

The PCR reaction system (20 μL) was configured as follows: 4 μL of 5× TransStart FastPfu Buffer (TransGen Biotech, Beijing, China), 2 μL of 2.5 mM dNTPs, 0.8 μL of each primer (5 μM), 0.4 μL of TransStart FastPfu DNA Polymerase, 10 ng of DNA template, and nuclease-free distilled water to adjust the volume. An equal volume of nuclease-free distilled water replaced the DNA template as the negative control. PCR conditions were set as follows: initial denaturation at 95 °C for 3 min, followed by 27 cycles of 95 °C for 30 s, 55 °C for 30 s, and 72 °C for 45 s. Amplified products were examined by 1% agarose gel electrophoresis and then sent to Shanghai Majorbio Bio-pharm Technology Co., Ltd. (Shanghai, China), for paired-end sequencing on the Illumina MiSeq PE300 platform (Illumina, Inc., San Diego, CA, USA).

All bioinformatic analyses were performed on the Majorbio Cloud platform [29]. After demultiplexing, paired-end raw reads were quality-filtered using fastp (v0.19.6), and the filtered reads were merged using FLASH (v1.2.7) [30,31]. The high-quality merged sequences were then denoised into amplicon sequence variants (ASVs) using the DADA2 algorithm implemented in QIIME2 (v2024.10) [32]. Taxonomic classification was performed against the SILVA 138 16S rRNA database using the classify-sklearn classifier with a confidence threshold of 70%. A detailed read tracking summary is provided in Supplementary Table S2.

### 2.4. Data Statistical Analysis

Metamorphosis and settlement rate, as well as the morphological indicators of the polyps (body length, body width, and tentacle length), were compared between the Control and SG44 groups using Student’s *t*-tests. Alpha diversity indices, including Chao, Ace, Sobs, Simpson, and Shannon indices, were calculated using MOTHUR (v1.30) to estimate bacterial community richness and diversity [33]. Differences in alpha diversity indices and in the relative abundances of dominant taxa among groups were evaluated using the Kruskal–Wallis test with false discovery rate (FDR) correction.

Beta diversity was visualized using non-metric multidimensional scaling (NMDS) based on Bray–Curtis distances, and the fit of the ordination was evaluated by the stress value and the R^2^ of the model. Differences in β-dispersion among the three groups were assessed based on Bray–Curtis dissimilarity using the PERMDISP analysis, followed by post-hoc comparisons using Tukey’s HSD test. Differences in community structure among the groups were further tested using analysis of similarities (ANOSIM) and permutational multivariate analysis of variance (ADONIS) based on Bray–Curtis distances.

The community composition at the genus level was summarized as the mean ± standard deviation (SD) of each group. Differences in the relative abundances of genera between the two groups were identified using the two-sided Wilcoxon rank-sum test with FDR correction.

For co-occurrence network analysis, ASVs present in at least five of the six samples within a group were retained for network construction, and ASVs with zero variance within a group were removed. Networks were constructed separately for each group using the NetCoMi package (v1.3.0) in R, with SparCC as the association measure, an approach designed for compositional microbiome data. Sparsification was performed by thresholding, retaining only edges with |r| ≥ 0.7, and self-loops were removed [34]. Network topological properties, including node degree, clustering coefficients, module structure, and eigenvector centrality-based hub identification, were calculated using the netAnalyze function. The networks were visualized using Gephi (v0.9.2) [35].

## 3. Results

### 3.1. Settlement and Metamorphosis Rate and Morphological Observations

**Figure 1:**
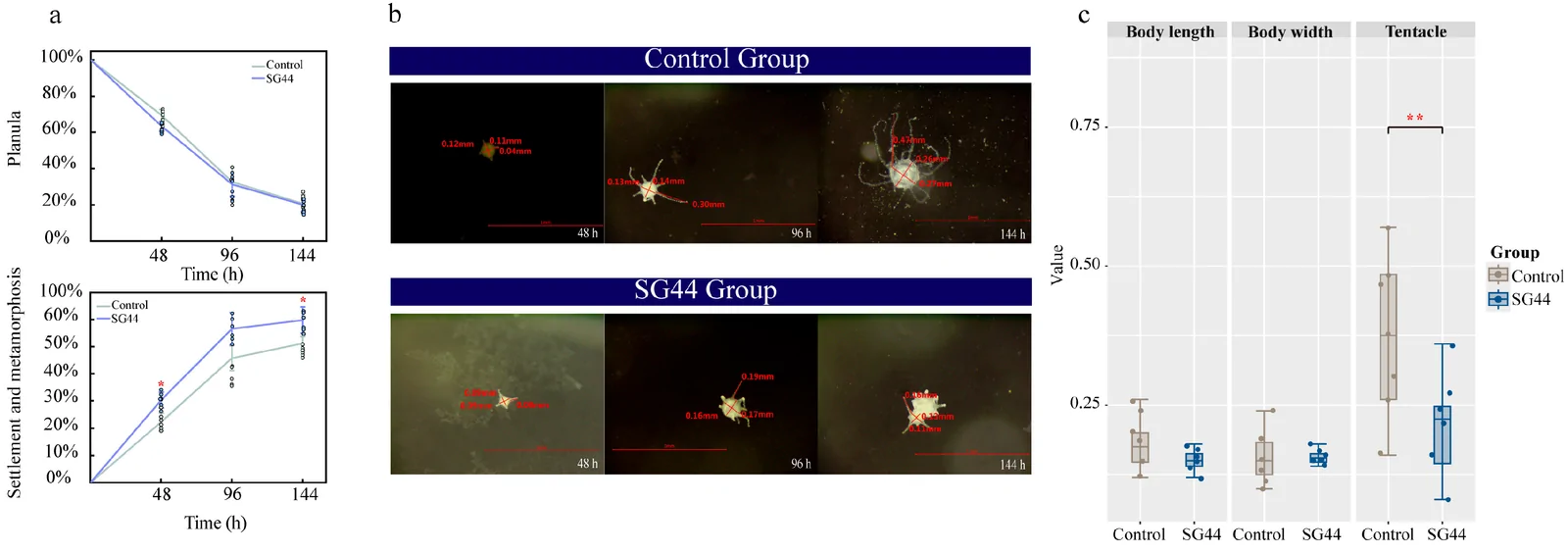
Effect of treatment with strain SG44 on the settlement and metamorphosis rate and morphology of *A. coerulea* planula larvae. *n* = 6. (**a**) Proportion of polyps and planulae in different groups. (**b**) Morphology of polyps. Scale bars, 1 mm. (**c**) Physiological indicators in Control Group and SG44 Group after 144 h. Differences in (**a**,**c**) between the two groups were assessed using Student’s *t*-test. *, *p* < 0.05; **, *p* < 0.01.

The settlement and metamorphosis rate of larvae, as well as the body length, body width, and tentacle length of polyps, were compared between the Control Group and the SG44 Group. The settlement and metamorphosis rate in the T Group was significantly higher than that in the Control Group after 48 h of culture (30.4 ± 2.9% and 22.4 ± 3.2%, respectively, Figure 1a). Despite initial similarities in larval morphology between the Control Group and SG44 Group, phenotypic distinctions became apparent during the cultivation period (Figure 1b). Final morphological measurements showed no significant differences in polyp body length or width between the two groups. However, the tentacle length of polyps in the SG44 Group was significantly reduced (Figure 1c). Furthermore, stereomicroscopic images notably confirmed severe developmental deformities in polyps of the SG44 Group.

### 3.2. Effect of Strain SG44 on Associated Microbial Diversity

**Figure 2:**
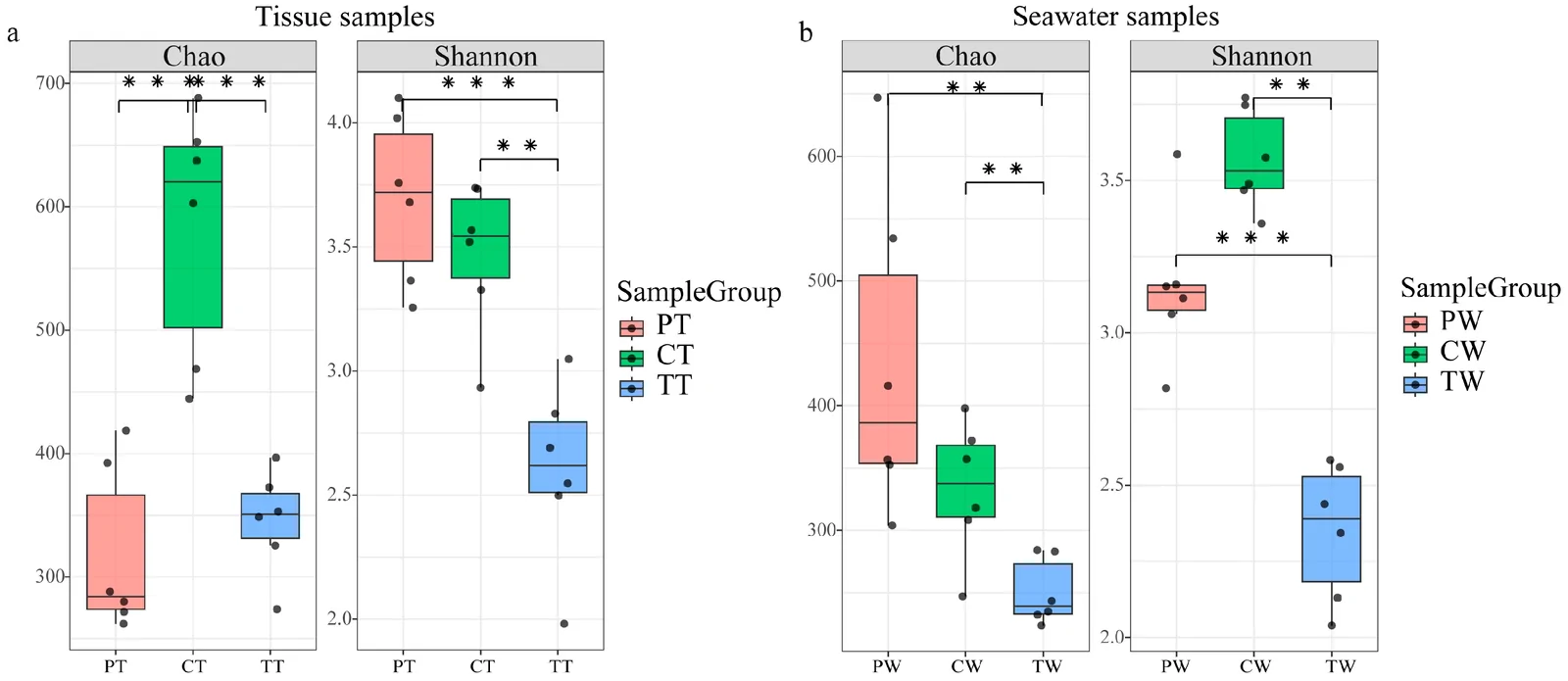
Alpha diversity indices (Chao and Shannon) of the associated microbial communities in (**a**) tissue samples and (**b**) seawater samples. *n* = 6. Dots represent individual data points. Statistical differences among the three groups were assessed using the Kruskal–Wallis test followed by pairwise comparisons with FDR correction. **, *p* adj < 0.01; ***, *p* adj < 0.001.

To investigate changes in the microbial community following co-culture with strain SG44, we sequenced the bacterial 16S rRNA genes from tissue samples and culture seawater samples. The samples included planula tissue (PT), control polyp tissue (CT), and treatment polyp tissue (TT). Corresponding culture seawater samples included planula seawater (PW), control seawater (CW), and treatment seawater (TW). A total of 2,080,275 raw reads were recovered from the 36 samples, yielding 1,553,177 high-quality reads after quality control (Table S2). These reads resulted in 3705 amplicon sequence variants (ASVs) from seawater and 4374 ASVs from tissue. The sample rarefaction curves exhibited a gradual increase followed by a plateau, suggesting that the current sequencing depth was sufficient and additional sequencing would not substantially change the observed species diversity (Figure S1). Analysis of alpha diversity revealed substantial changes in the microbial community’s diversity within polyps following treatment with strain SG44. Compared to CT (582.40 ± 101.54 and 3.47 ± 0.30), the Chao and Shannon indices of TT were significantly lower (345.04 ± 42.32 and 2.60 ± 0.36, respectively) (Figure 2a, Table S3). A similar trend was observed in culture seawater samples; the Chao and Shannon indices of TW (250.21 ± 26.57 and 2.35 ± 0.22) were significantly lower than those of CW (333.39 ± 58.87 and 3.57 ± 0.16) (Figure 2b, Table S4). Consistently, the ACE and Simpson indices also supported this finding (Figure S3, Tables S3 and S4).

### 3.3. Alteration of Microbial Community Composition by Strain SG44

**Figure 3:**
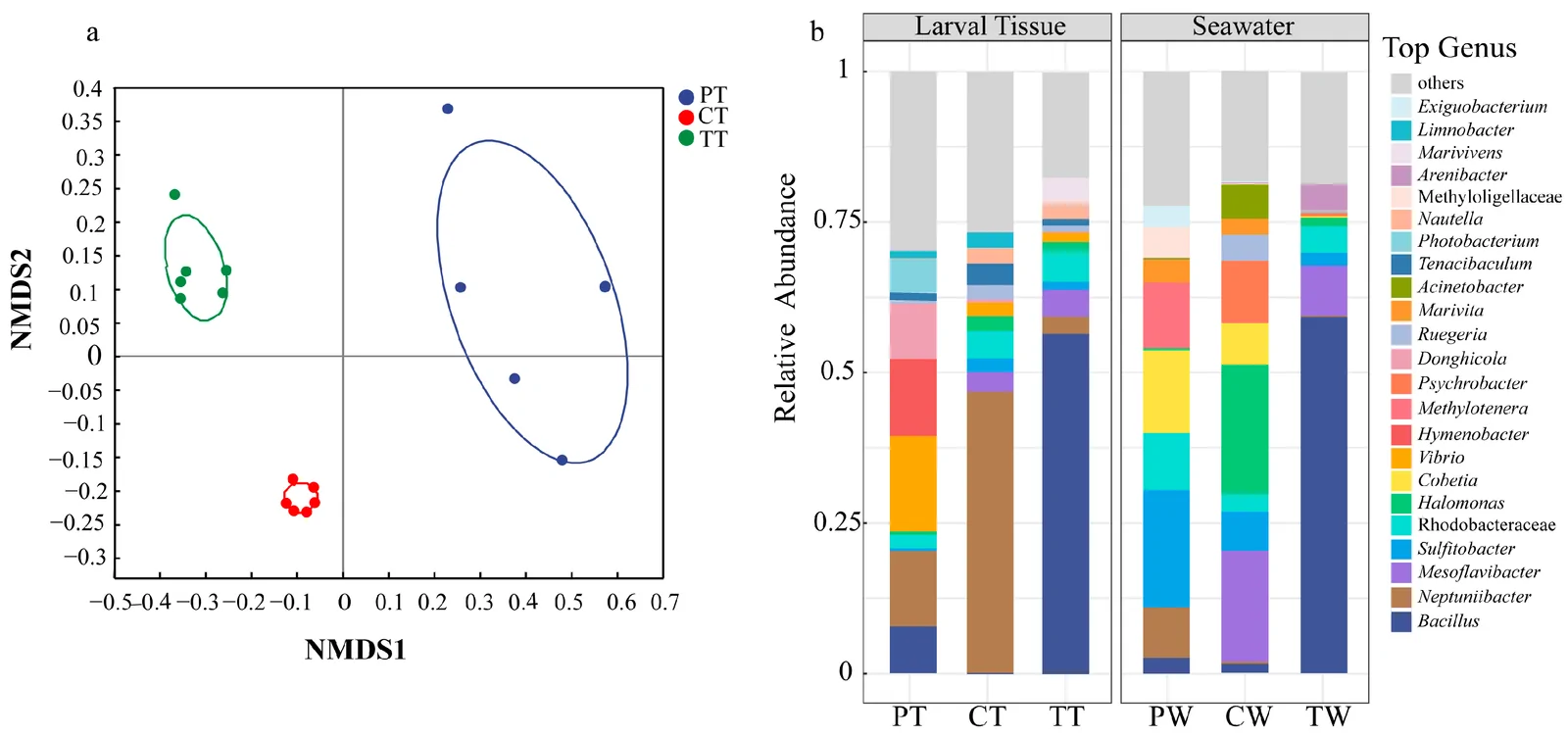
Associated microbial communities of newly released planulae, polyp tissues, and culture seawater. *n* = 6. (**a**) NMDS of epibiotic microorganisms in tissue samples from groups P, C and T (stress = 0.083, R^2^ = 0.6874, P = 0.001). (**b**) Bacterial community composition at the genus level.

Non-metric multidimensional scaling (NMDS) based on Bray–Curtis distances revealed a clear separation of microbial communities among PT, CT, and TT (Figure 3a), and a similar pattern was observed for the seawater samples (Figure S2). PERMDISP analysis indicated significant differences in multivariate dispersion among groups, with markedly higher within-group heterogeneity in PT than in the more homogeneous CT and TT (Figure S4a), and significantly lower dispersion in TW than in PW and CW (Figure S4b). Nevertheless, both ANOSIM and ADONIS consistently revealed significant differences in community composition among PT, CT, and TT (Table S5), and among the seawater communities PW, CW, and TW (Table S5). Together with the NMDS patterns, these results indicate that the host-associated microbiota was substantially restructured during metamorphosis from the planula to the polyp stage and was further altered after treatment with strain SG44 (Figure 3b).

**Figure 4:**
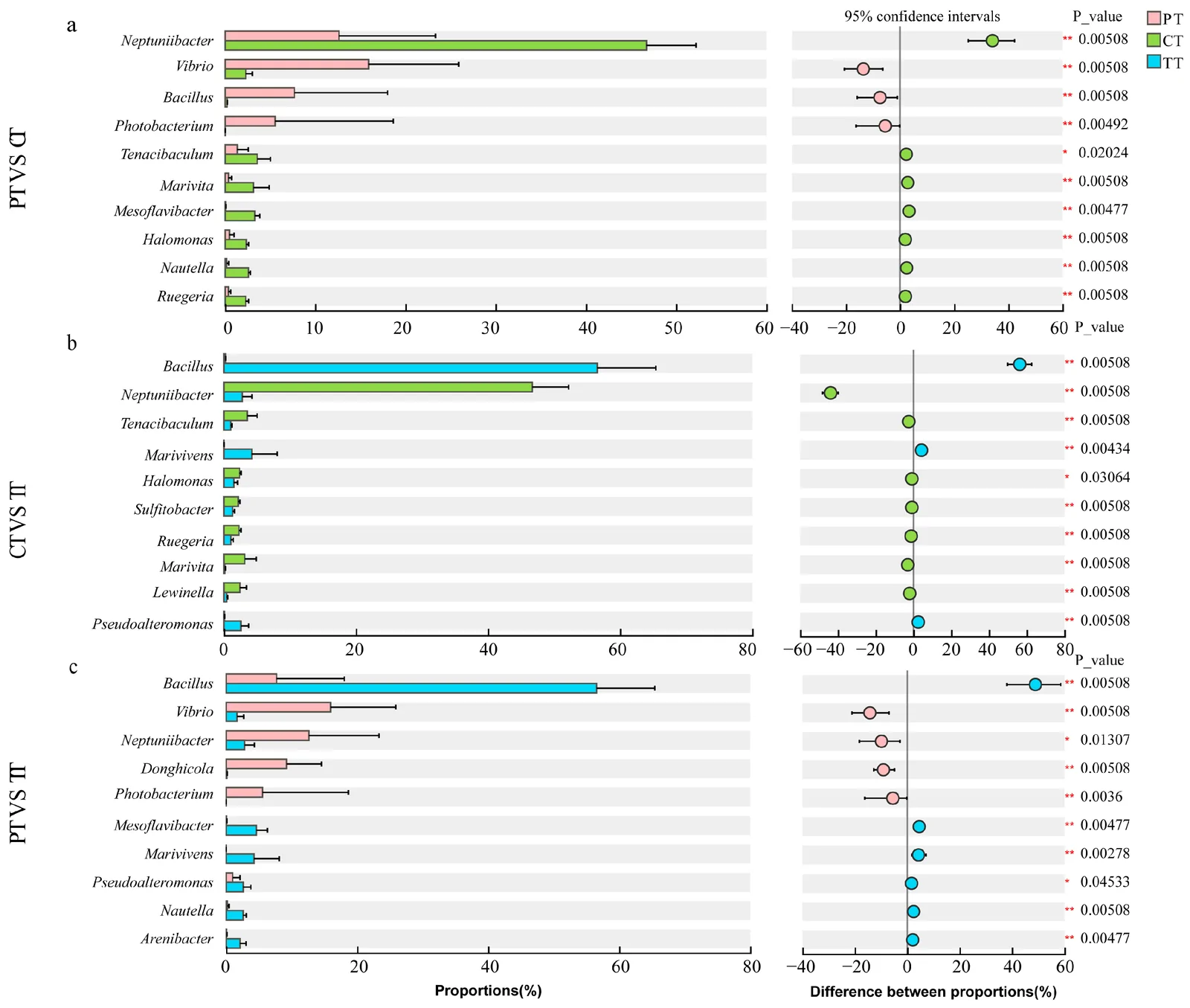
Pairwise comparisons of differences in associated microbial communities among tissue samples from groups P, C, T. *n* = 6. (**a**) PT vs. CT; (**b**) CT vs. TT; (**c**) PT vs. TT. (*Y*-axis: differentially abundant genera; left *x*-axis: mean relative abundance (%); right *x*-axis: difference in mean relative proportions between the two compared groups). Differential abundance was tested by two-sided Wilcoxon rank-sum tests with FDR correction. *, *p* adj < 0.05, **, *p* adj < 0.01.

These changes in microbial community structure suggested that the developmental transition of *A. coerulea* from planula to polyp stage was accompanied by significant changes in microbial community composition. In PT, the most abundant genera were Vibrio (15.9 ± 10.0%), *Hymenobacter* (12.8 ± 18.7%), and *Neptuniibacter* (12.7 ± 10.7%) (Figure 3b). In CT, *Neptuniibacter* was the dominant genus, accounting for 46.71 ± 5.50% of the microbial community, followed by unclassified *Rhodobacteraceae* (4.6 ± 0.4%) and *Tenacibaculum* (3.5 ± 1.4%) (Figure 3b). Treatment with strain SG44 substantially altered the polyp-associated microbiota. In TT, *Bacillus* became the predominant genus, comprising 56.5 ± 8.9% (Figure 3b) of the total population. Compared to CT, the abundances of *Halomonas* (2.37 ± 0.26% vs. 1.52 ± 0.54%), *Sulfitobacter* (2.16 ± 0.30% vs. 1.31 ± 0.30%), and *Ruegeria* (2.30 ± 0.33% vs. 1.05 ± 0.38%) were significantly reduced in TT (Figure 4). Notably, the relative abundance of *Pseudoalteromonas* (CT: 0.71 ± 0.05% vs. TT: 2.6 ± 1.15%) increased significantly in TT (Figure 4). In seawater samples, *Bacillus* was the dominant microbial genus in TW, with a significantly higher relative abundance than in CW (*p* < 0.05). Conversely, the relative abundance of *Halomonas* (TW: 1.4 ± 0.6%, CW: 21.4 ± 17.2%), *Psychrobacter* (TW: 0.6 ± 0.5%, CW: 10.2 ± 18.0%), *Sulfitobacter* (TW: 2.1 ± 1.5%, CW: 6.4 ± 2.1%), *Cobetia* (TW: 0.2 ± 0.4%, CW: 7.0 ± 7.5%), and *Ruegeria* (TW: 0.5 ± 0.1%, CW: 4.4 ± 2.5%) were significantly lower in TW (Figure 3b). Compared to PW, the relative abundance of *Neptuniibacter* in CW was significantly reduced (*p* < 0.05) (Figure S5). Collectively, the changes in microbial community of both tissue and seawater samples indicated successful metamorphosis of planulae to polyps during culture, and the microbial community structure of polyps in the SG44 treatment was significantly altered. Notably, the dominant ASV in both TW and TT, ASV1, was classified as *Bacillus pumilus*, consistent with the taxonomic identity of strain SG44.

### 3.4. Microbial Community Co-Occurrence Network Analysis

**Figure 5:**
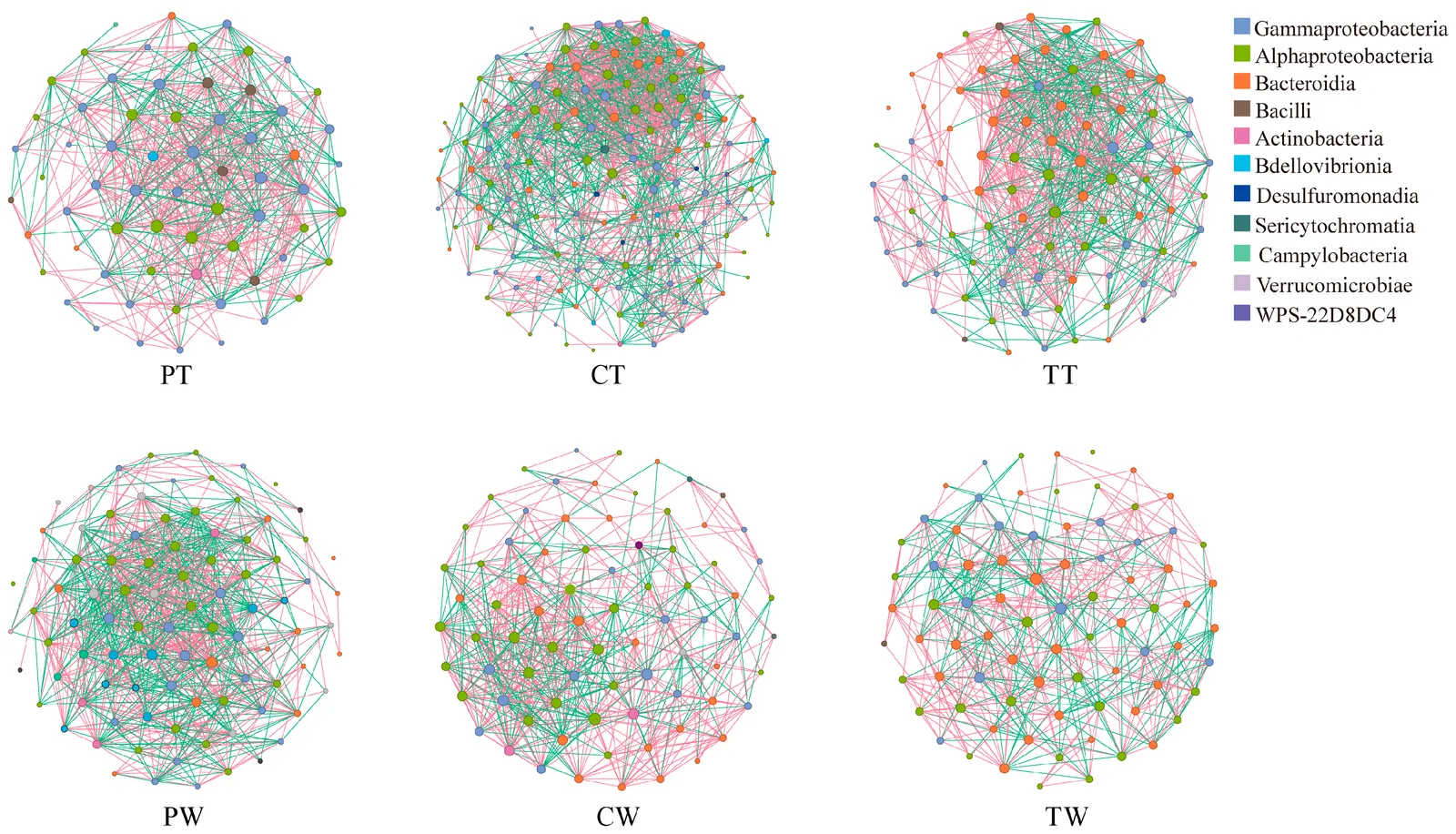
Associated microbial community co-occurrence networks at the ASV level for newly released planulae, polyp tissues, and culture seawater samples. *n* = 6. (ASVs present in at least five of the six samples within each group were retained for network construction. Node colors represent different classes, and node size is proportional to node degree. Red edges indicate positive correlations, whereas green edges indicate negative correlations).

Co-occurrence networks were constructed to characterize co-occurrence patterns among microbial taxa. In the tissue networks, the hub class shifted across developmental stages and treatments: the networks in PT and CT were dominated by Gammaproteobacteria and Alphaproteobacteria, respectively, whereas in TT a marked shift was observed, wherein Bacteroidia accounted for the largest number of nodes (Figure 5). A parallel shift in hub classes was observed in the seawater networks (Figure 5). Network topology varied substantially across developmental stages and across the treatments. During normal development from PT to CT, the network expanded in size, with nodes increasing from 65 to 151 and edges from 610 to 1486, and became more densely connected, as indicated by an increase in average degree from 18.76 to 19.68. Meanwhile, the proportion of positive correlations decreased from 62.79% to 53.36%, while negative correlations increased from 37.21% to 46.64% (Table S6). In contrast, in TT, the network contracted in size, with nodes and edges decreasing to 90 and 898, respectively, but maintained a comparably high average degree (19.96) and a higher proportion of positive correlations than in the control polyps (59.24% vs. 53.36%) (Table S6). To determine whether the observed changes in the TT network were caused by sequencing bias associated with the exogenous strain SG44 (ASV1), we also constructed co-occurrence networks for TT after removing ASV1. The results showed that, compared with CT, the network differences in TT persisted and followed the same trend (Table S6).

## 4. Discussion

Microorganisms are important partners that play crucial roles in the growth and development of marine invertebrates [36]. In microbe–host interactions, microbes not only offer essential help for host nutrient acquisition and biogeochemical cycling [37,38], but they also help hosts adapt to niche variation and enhance resilience against environmental stressors by altering their community structure or function [39]. Alterations in host microbial composition, such as shifts in taxa, relative abundances, or the absence of native groups, indicate changes in host adaptability [40]. We investigated the effect of bacterial strain SG44 on the settlement and metamorphosis of *A. coerulea* planula larvae. Our findings indicated that strain SG44 disrupted the normal settlement and metamorphosis process of planulae, resulting in elevated settlement and metamorphosis rate coupled with abnormal polyp morphology, which coincided with alterations in the microbial community structure and disruption of host-associated microbial interactions.

The settlement and metamorphosis of planula larvae are critical stages in the jellyfish life cycle [41]. Our results showed that although treatment with strain SG44 increased the settlement and metamorphosis rate of planulae, the resulting polyps exhibited abnormal morphology. This marked elevation in metamorphosis rate may be explained by the “Desperate Larva Hypothesis”, which proposes that under perceived survival threat, larvae reduce their selectivity for settlement and metamorphosis cues and accelerate development to shorten the vulnerable planktonic phase, thereby enhancing survival probability [42]. The introduction of the exogenous microorganism SG44 may induce an adverse, stressful microenvironment for the planulae, thereby eliciting this adaptive stress response. This mechanism aligns with developmental strategies observed under other stressors; for example, elevated temperature accelerates larval development and shortens the planktonic duration of many marine invertebrates, reducing the time larvae spend in the vulnerable dispersal phase [43]. Therefore, the increased settlement and metamorphosis rate, coupled with the observed morphological abnormality, should not be regarded as a beneficial effect of strain SG44 on the population recruitment of *Aurelia coerulea*. As tentacles serve as the primary feeding apparatus, this structural aberration directly impacts prey capture efficiency, which in turn adversely impacts the organism’s ability to acquire energy [44,45]. Due to impaired feeding and morphological defects, polyps may adopt a conservative energy allocation strategy, directing limited resources preferentially towards sustaining basal life functions and metabolic homeostasis [44]. This shift in energy budgeting could disrupt developmental progression. Notably, such resource limitation prevents the initiation of strobilation, leading to a decreased output of ephyrae [46]. Collectively, these potential downstream effects may constrain population recruitment in *A. coerulea*. However, direct measurements of feeding performance, energy reserves, and strobilation output are needed to validate these inferences.

The treatment with strain SG44 significantly altered the microbial communities in both the polyps and the surrounding culture seawater. Compared to the control group dominated by *Neptuniibacter*, *Bacillus* became dominant in the treatment group, with significant decreases in *Halomonas*, *Sulfitobacter*, and *Ruegeria* abundances. There is evidence that *Neptuniibacter* is involved in host–microbe interactions and is a potential probiotic assisting jellyfish in normal growth and development [3]. In marine invertebrate microbiomes, *Halomonas*, *Sulfitobacter*, and *Ruegeria* are common and functionally important core taxa. They positively influence host growth and development by supporting metabolic functions, regulating immune responses, and facilitating signal transduction. *Halomonas* enhances host immune function and assists its nutrient metabolism through synergistic effects, providing crucial support for healthy host growth and stress resistance [47,48,49]. *Sulfitobacter* and *Ruegeria* can regulate host immune signalling pathways and synthesize various antioxidant substances to enhance host stress resistance and defense capabilities [50,51]. Additionally, *Ruegeria* can secrete quorum-sensing signal molecules that are involved in larval settlement and metamorphosis [52]. Notably, all three can effectively inhibit the colonization of pathogens such as *Vibrio coralliilyticus* and *Vibrio harveyi* by competing for ecological niches and nutrients, as well as secreting antimicrobial peptides and secondary metabolites that maintain a microecological balance within the host [53,54]. As a result of the suppression of probiotics such as *Neptuniibacter*, *Halomonas*, *Sulfitobacter*, and *Ruegeria*, coupled with a disruption of microbial cooperation, the host may have experienced immune and metabolic imbalances; however, whether these imbalances contributed to the observed polyp deformities remains to be determined. These observations suggest that the microbiota restructuring observed following SG44 exposure may be associated with the observed developmental abnormalities and abnormal larval settlement and metamorphosis, although a causal relationship remains to be established.

Furthermore, co-occurrence network analysis may offer additional, albeit tentative, insights into the ecological implications of the observed shifts in community structure. In the control polyp tissue, the network expanded considerably in size from planula to polyp and became more densely connected, accompanied by a decrease in the proportion of positive correlations. It is plausible that, under normal conditions, the development from planula to polyp incorporates more taxa into the co-occurrence network and intensifies their associations, accompanied by a shift in the balance between positive and negative correlations, a pattern that could reflect micro-ecological reorganization during host maturation [55,56]. In striking contrast, the network of treatment polyp tissue exhibited a fundamentally different architecture. Despite a substantially reduced number of nodes, consistent with the decline in microbial diversity following treatment with strain SG44, the network maintained a comparably high average degree and a higher proportion of positive correlations. In parallel, the community shift after treatment also showed signs of oligarchizing, with a marked loss of evenness such that a small number of lineages came to dominate the community [57,58]. Therefore, the network associated with treatment by strain SG44 appears to represent a configuration distinct from that of the control. However, co-occurrence networks reflect statistical associations rather than direct ecological interactions, and given the inherent limitations of network inference based on *n* = 6 samples, we positioned this analysis as exploratory; the observed network differences should not be interpreted as direct ecological effects of strain SG44, and any ecological interpretation remains speculative pending functional validation. These considerations are compatible with the polyp deformities observed in this study, although a causal link remains to be established. It should be noted that strain SG44 used in this study, which belongs to *Bacillus pumilus*, is a common non-pathogenic bacterium widely distributed in marine and terrestrial environments and has been extensively employed as a probiotic in aquaculture [59,60]. The effect of this strain on larval settlement and metamorphosis may involve indirect mechanisms, such as changes in the host-associated microbial community, rather than direct cytotoxicity; however, the relative contributions of these possible pathways are currently unclear. Meanwhile, one limitation of this study is that physicochemical parameters of the rearing water were not measured; therefore, potential changes in water chemistry caused by the treatment with strain SG44 cannot be ruled out as a contributing factor to the observed differences between CW and TW.

## 5. Conclusions

This study investigated the effects of strain SG44 on the bacterial communities associated with *Aurelia coerulea* planula during settlement and metamorphosis. Co-culture with strain SG44 significantly increased larval settlement and metamorphosis rate, while the resulting polyps exhibited morphological deformities, predominantly shortened tentacles. Meanwhile, the polyp-associated bacterial community and its co-occurrence network were markedly altered: microbial diversity decreased, and the co-occurrence network contracted in size while maintaining a comparably high average degree. Strain SG44 became established within the polyp-associated community, accompanied by a reduced abundance of core taxa including *Neptuniibacter* and *Halomonas*. Based on these observations, we hypothesize that the alterations in the host microbial community and co-occurrence network patterns after treatment with strain SG44 may be associated with abnormal larval settlement and metamorphosis. However, the causal relationship and underlying mechanisms remain to be elucidated. These findings provide insights into microbe–host interactions during larval settlement and metamorphosis in scyphozoans.

## Data Availability

Raw reads of the 16S rRNA gene generated in this work were deposited in the NCBI sequence read archive under the BioProject accession number PRJNA1297361 (https://www.ncbi.nlm.nih.gov/bioproject/PRJNA1297361, accessed on 12 October 2026).

## Supplementary Materials

The following supporting information can be downloaded at: https://www.mdpi.com/article/10.3390/microorganisms14102338/s1, Figure S1: Rarefaction curves of associated microbial communities (PT: planulae tissue; CT: control tissue; TT: SG44-treated tissue). Figure S2: NMDS of bacterial communities in planula seawater (PW), control seawater (CW), and SG44-treated culture seawater (TW). Figure S3: Alpha diversity indices (ACE and Simpson) of the associated microbial communities in tissue samples (a) and seawater samples (b). *n* = 6. Statistical differences among the three groups were assessed using the Kruskal–Wallis rank-sum test followed by pairwise post-hoc comparisons with FDR correction. Each box plot represents *n* = 6 biological replicates. *, *p* < 0.05; **, *p* < 0.01; ***, *p* < 0.001. Figure S4: Beta dispersion of the associated microbial communities in polyp tissue samples (a) and seawater samples (b). *n* = 6. Dispersion represents Bray–Curtis dissimilarity of each sample relative to its group centroid. Statistical differences in dispersion among the three groups were assessed using PERMDISP (999 permutations) followed by pairwise comparisons with Tukey’s HSD correction. Groups with different letters indicate significant differences (*p* adj < 0.05). Figure S5: Pairwise comparisons of differences in associated microbial communities among seawater samples from groups P, C, T. *n* = 6. (*Y*-axis: differentially abundant genera; left *x*-axis: mean relative abundance (%); right *x*-axis: difference in mean relative proportions between the two compared groups). Differential abundance was tested by two-sided Wilcoxon rank-sum tests with FDR correction. *, *p* adj < 0.05, **, *p* < 0.01, ***, *p* < 0.001. Table S1: Experimental groups and their nomenclature. Table S2: Sequencing depth and quality control summary for all samples. Table S3: Alpha diversity indices of the bacterial communities associated with *Aurelia coerulea* planulae and polyps. Table S4: Alpha diversity indices of bacterial communities in culture seawater associated with *Aurelia coerulea*. Table S5: Results of ADONIS and ANOSIM tests for three sample groups. Table S6: Characteristics of the co-occurrence network of associated microorganisms in different treatment tissues and cultured seawater.
