# Supplementary material for: Bacillus pumilus SG44 Reshapes the Microbiome and Alters Metamorphosis and Development of Aurelia coerulea Polyps
Source: Microorganisms. 2026 Oct 13;14(10):2338. doi: 10.3390/microorganisms14102338 (PMC13707911; doi:10.3390/microorganisms14102338)
Supplement: Supplementary file 1 [file microorganisms-14-02338-s001.zip › microorganisms-4558757-supplementary.pdf]

Bacillus pumilus SG44 Reshapes the Microbiome and Alters Metamorphosis and Development of *Aurelia coerulea* Polyps

Min Li [1], Xiangrui Guo [1], Manzoor Ahmad [2], Jiajing Yang [1], Jie Sun [3], Zirui Li [4], Hongyuan Lin [1], Yuting Zhang [1], Ye Zhao [1], Hao Sun [1] and Yanying Zhang [1,*]

[1] School of Ocean, Yantai University, Yantai 264005, China; lmin1821@s.ytu.edu.cn (M.L.)
[2] Department of Integrative Agriculture, College of Agriculture and Veterinary Medicine, United Arab Emirates University, Al Ain 200120, United Arab Emirates
[3] Fisheries College, Ocean University of China, Qingdao 266003, China
[4] College of Marine Sciences, South China Agricultural University, Guangzhou 510642, China
* Correspondence: zhyanying@ytu.edu.cn

Supplementary Figures

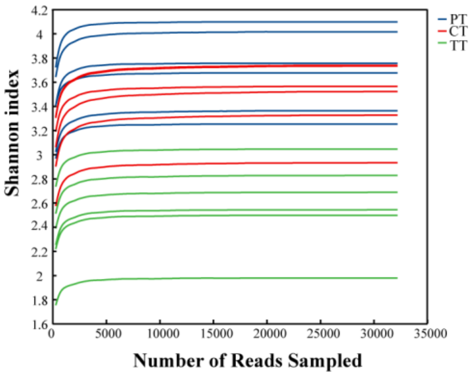

Figure S1. Rarefaction curves of associated microbial communities (PT: planulae tissue; CT: control tissue; TT: SG44-treated tissue).

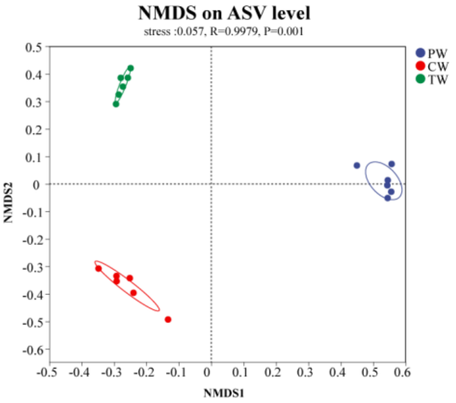

Figure S2. NMDS of bacterial communities in planula seawater (PW), control seawater (CW), and SG44-treated culture seawater (TW).

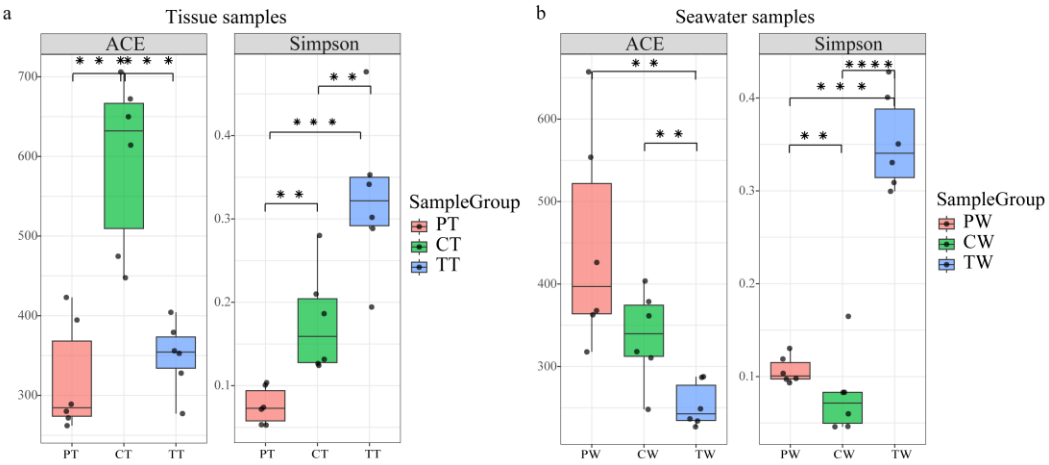

Figure S3. Alpha diversity indices (ACE and Simpson) of the associated microbial communities in tissue samples (a) and seawater samples (b). n=6. Statistical differences among the three groups were assessed using the Kruskal-Wallis rank-sum test followed by pairwise post-hoc comparisons with FDR correction. Each box plot represents n = 6 biological replicates. *, $P < 0.05$; **, $P < 0.01$; ***, $P < 0.001$.

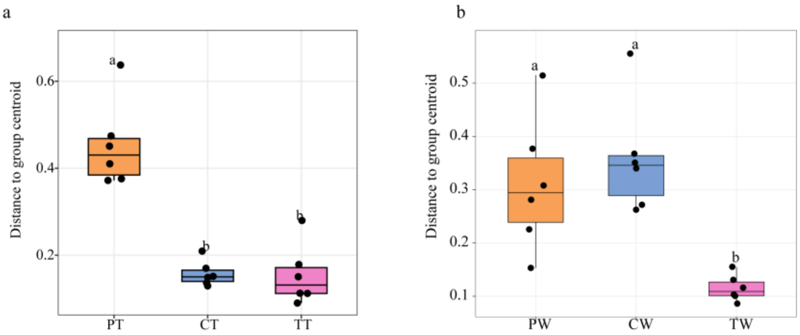

Figure S4. Beta dispersion of the associated microbial communities in polyp tissue samples (a) and seawater samples (b). n=6. Dispersion represents Bray-Curtis dissimilarity of each sample relative to its group centroid. Statistical differences in dispersion among the three groups were assessed using PERMDISP (999 permutations) followed by pairwise comparisons with Tukey's HSD correction. Groups with different letters indicate significant differences (P adj < 0.05).

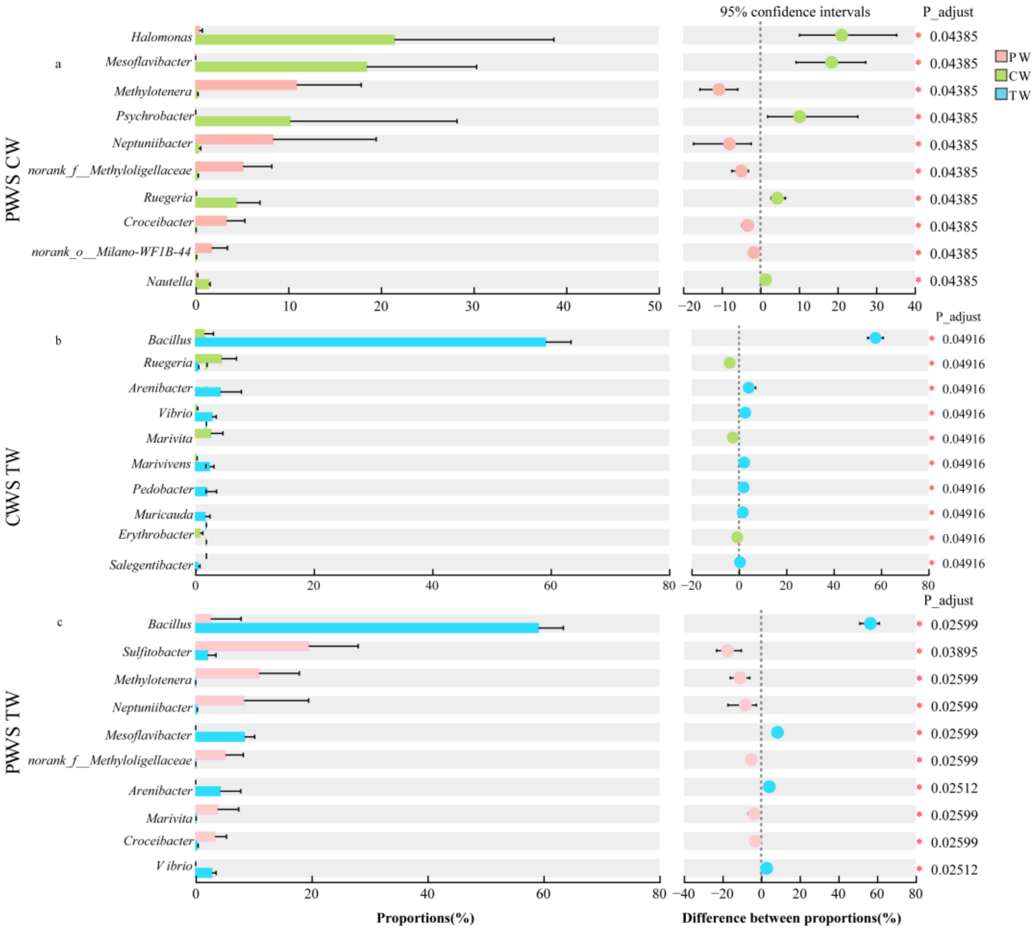

Figure S5. Pairwise comparisons of differences in associated microbial communities among seawater samples from groups P, C, T. n=6. (Y-axis: differentially abundant genera; left x-axis: mean relative abundance (%); right x-axis: difference in mean relative proportions between the two compared groups). Differential abundance was tested by two-sided Wilcoxon rank-sum tests with FDR correction. *, P adj < 0.05, **, P < 0.01, ***, P < 0.001.

Supplementary Tables

Table S1. Experimental groups and their nomenclature

Group ID	Group description	Sample type	Duration	n
PT	Planulae	Tissue	0 h	6
PW	Planulae	Seawater	0 h	6
CT	Control	Tissue	144 h	6
CW	Control	Seawater	144 h	6
TT	Treatment with strain SG44	Tissue	144 h	6
TW	Treatment with strain SG44	Seawater	144 h	6

Table S2. Sequencing depth and quality control summary for all samples.

Sample	Number of reads	Reads after QC	% reads based QC	% reads identified (genus level)
PT1	74,330	64,779	87.15%	91.98%
PT2	45,940	40,899	89.03%	90.77%
PT3	52,596	45,424	86.36%	96.60%
PT4	40,843	32,194	78.82%	95.25%
PT5	114,845	90,725	79.00%	94.87%
PT6	49,963	38,314	76.68%	90.10%
PW1	58,001	45,600	78.62%	75.27%
PW2	82,425	59,506	72.19%	77.26%
PW3	57,791	46,667	80.75%	72.66%
PW4	64,547	48,534	75.19%	86.32%
PW5	60,455	48,179	79.69%	80.64%
PW6	49,184	39,877	81.08%	60.84%
CT1	48,819	34,696	71.07%	85.51%
CT2	54,108	38,439	71.04%	91.08%
CT3	63,249	41,615	65.80%	88.12%
CT4	66,874	43,455	64.98%	89.41%
CT5	67,675	44,376	65.57%	85.68%
CT6	69,000	45,288	65.63%	84.17%
CW1	52,613	33,041	62.80%	94.83%
CW2	52,362	41,969	80.15%	88.79%
CW3	58,025	41,416	71.38%	94.02%
CW4	57,291	37,295	65.10%	93.07%
CW5	57,246	36,303	63.42%	95.31%
CW6	49,124	32,129	65.40%	94.77%
TT1	49,295	35,872	72.77%	84.61%
TT2	48,099	37,055	77.04%	95.23%
TT3	45,041	35,812	79.51%	96.62%
TT4	55,657	41,569	74.69%	91.88%
TT5	55,944	42,489	75.95%	91.80%

TT6	52,874	40,532	76.66%	91.98%
TW1	49,314	38,388	77.84%	93.89%
TW2	52,051	41,789	80.28%	93.05%
TW3	48,360	36,354	75.17%	94.70%
TW4	51,998	38,808	74.63%	92.67%
TW5	59,082	44,417	75.18%	94.48%
TW6	65,254	49,372	75.66%	96.11%

Table S3. Alpha diversity indices of the bacterial communities associated with *Aurelia coerulea* planulae and polyps

Samples	ACE	Chao	Shannon	Simpson	Coverage
PT1	271.7088	271.75	3.758483	0.074034	99.99%
PT2	262	262	4.099986	0.052999	100.00%
PT3	288.9354	288.05	3.679936	0.052382	99.98%
PT4	280	280	3.255716	0.103628	100.00%
PT5	423.0066	418.7143	3.364516	0.100466	99.91%
PT6	394.6561	392.4865	4.019427	0.071585	99.97%
CT1	447.6412	444.5769	3.567234	0.131664	99.97%
CT2	474.6442	468.5584	2.932733	0.280168	99.92%
CT3	705.95	688.2574	3.520309	0.186414	99.79%
CT4	672.2328	652.7143	3.327138	0.21013	99.80%
CT5	649.8516	637.4286	3.733802	0.126251	99.79%
CT6	614.193	602.9159	3.737926	0.124422	99.84%
TT1	355.9501	353.0377	3.047888	0.194446	99.97%
TT2	327.9329	325.5	2.498775	0.352791	99.98%
TT3	277.117	273.814	1.981243	0.4767	99.96%
TT4	404.2801	396.6418	2.828644	0.288522	99.91%
TT5	379.1341	372.6111	2.691055	0.302086	99.90%
TT6	352.7626	348.6154	2.546707	0.3415	99.95%

Table S4. Alpha diversity indices of bacterial communities in culture seawater associated with *Aurelia coerulea*

Samples	ACE	Chao	Shannon	Simpson	Coverage
PW1	426.1158	415.859	3.158059	0.093378	99.85%
PW2	553.6679	534.2039	3.152134	0.097899	99.70%
PW3	367.738	356.7011	3.061469	0.103412	99.90%
PW4	317.7052	303.8871	3.113853	0.097169	99.87%
PW5	362.622	352.7377	2.818336	0.130382	99.84%
PW6	657.1923	647.0513	3.585814	0.11897	99.85%
CW1	247.981	247.075	3.468511	0.059911	99.99%
CW2	403.6057	397.8077	3.358732	0.164896	99.91%
CW3	378.6279	371.9531	3.575149	0.082918	99.93%
CW4	361.391	357.1053	3.771534	0.046312	99.95%
CW5	310.4608	308.4118	3.488851	0.083094	99.98%
CW6	318	318	3.747275	0.045994	100.00%
TW1	236.4666	234.913	2.438209	0.330422	99.98%
TW2	226.9046	223.6667	2.129995	0.400632	99.97%
TW3	233.9189	232.2778	2.342936	0.350605	99.98%
TW4	287.6508	284.0769	2.582228	0.308854	99.95%
TW5	286.7626	283	2.559002	0.299512	99.94%
TW6	248.6971	243.3235	2.039413	0.42815	99.92%

Table S5. Results of ADONIS and ANOSIM tests for three sample groups

Group	Test method	Statistic name	Statistic value	Value
PT VS CT	ADONIS	R^2	0.856215	0.003
	ANOSIM	R	0.916667	0.003
CT VS TT	ADONIS	R^2	0.528507	0.003
	ANOSIM	R	1	0.003
PT VS TT	ADONIS	R^2	0.550159	0.003
	ANOSIM	R	0.955556	0.003
PW VS CW	ADONIS	R^2	0.5548	0.003
	ANOSIM	R	1	0.003
CW VS TW	ADONIS	R^2	0.6482	0.003
	ANOSIM	R	0.9907	0.003
PW VS TW	ADONIS	R^2	0.7569	0.003
	ANOSIM	R	1	0.003

Table S6. Characteristics of the co-occurrence network of associated microorganisms in different treatment tissues and cultured seawater

	Tissue				Seawater		
Properties	PT	CT	TT	TT (No ASV1)	PW	CW	TW
nodes	65	151	90	89	91	78	79
edges	610	1486	898	871	980	598	509
Positive correlations	62.79%	53.36%	59.24%	58.78%	51.63%	63.38%	60.71%
Negative correlations	37.21%	46.64%	40.76%	41.22%	48.37%	36.62%	39.29%
Average degree	18.769	19.682	19.956	19.573	21.538	15.333	12.886
Hub class	Gammaproteobacteria	Alphaproteobacteria	Bacteroidia	Bacteroidia	Gammaproteobacteria	Alphaproteobacteria	Bacteroidia

Note: TT (No ASV1) denotes networks constructed after removing ASV1 from the input table.
